# Detecting space–time patterns of disease risk under dynamic background population

**DOI:** 10.1007/s10109-022-00377-7

**Published:** 2022-04-20

**Authors:** Alexander Hohl, Wenwu Tang, Irene Casas, Xun Shi, Eric Delmelle

**Affiliations:** 1 Department of Geography, University of Utah, Salt Lake City, UT 84112 USA; 2 Center for Applied Geographic Information Science, University of North Carolina at Charlotte, Charlotte, NC 28223 USA; 3 Department of Geography and Earth Sciences, University of North Carolina at Charlotte, Charlotte, NC 28223 USA; 4 School of History and Social Sciences, Louisiana Tech University, Ruston, LA 71272 USA; 5 Department of Geography, Dartmouth College, Hanover, NH 03755 USA; 6 Department of Geographical and Historical Studies, University of Eastern Finland, Joensuu Campus, P.O. Box 111, FI-80101 Finland

**Keywords:** Kernel density estimation, Dengue, Background, Adaptive, Population, C21, C63

## Abstract

We are able to collect vast quantities of spatiotemporal data due to recent technological advances. Exploratory space–time data analysis approaches can facilitate the detection of patterns and formation of hypotheses about their driving processes. However, geographic patterns of social phenomena like crime or disease are driven by the underlying population. This research aims for incorporating temporal population dynamics into spatial analysis, a key omission of previous methods. As population data are becoming available at finer spatial and temporal granularity, we are increasingly able to capture the dynamic patterns of human activity. In this paper, we modify the space–time kernel density estimation method by accounting for spatially and temporally dynamic background populations (ST-DB), assess the benefits of considering the temporal dimension and finally, compare ST-DB to its purely spatial counterpart. We delineate clusters and compare them, as well as their significance, across multiple parameter configurations. We apply ST-DB to an outbreak of dengue fever in Cali, Colombia during 2010–2011. Our results show that incorporating the temporal dimension improves our ability to delineate significant clusters. This study addresses an urgent need in the spatiotemporal analysis literature by using population data at high spatial and temporal resolutions.

## Introduction

Novel methodologies for spatial and temporal analysis of geographic phenomena have emerged due to an abundance of geospatial information at the individual level (Anselin 2011). Domain examples include crimes (Wang et al. 2017; Malleson and Andresen 2015a; Koo et al. 2020) and disease events (Hohl et al. 2016; Delmelle et al. 2014), which typically cluster near city centers or exhibit seasonal cyclic patterns. Knowledge about the intensity, scale, location, and time of such clusters is important. In the case of disease outbreaks, this information is critical to inform authorities on their decision to allocate resources, such as staff for disease prevention efforts (Casas et al. 2010). Spatial and spatiotemporal statistics are a set of popular analytical methods for identifying and quantifying inherent patterns in the data, as they capture geospatial phenomena and their variability in space and time (Bailey and Gatrell 1995; Cressie and Wikle 2015) and across multiple scales (Fotheringham et al. 2017). Among the palette of exploratory statistics used to characterize a given spatiotemporal point pattern, space–time kernel density estimation (STKDE; Nakaya and Yano 2010) stands out. It allows for visualizing the occurrence of events in space and time by computing the localized intensity of the point process at hand and hence, summarizing the distribution of a spatial variable through time. STKDE has been employed as a key analytical procedure for identifying clusters of crime (Nakaya and Yano 2010), exploring human mobility patterns (Gao 2015), as well as discovering outbreaks of dengue fever (Delmelle et al. 2014).

Disease risk can be understood as the ratio between the number of disease cases and the population-at-risk within a given area (Desjardins et al. 2020). Therefore, it is imperative to consider density estimates of disease cases in relation to the local population-at-risk (a.k.a. ‘the background’). Otherwise, the estimates are a proxy of the population distribution and we might observe a cluster of high density merely due to a large local population. While conventional STKDE does not consider the background at all, several approaches incorporate spatially varying backgrounds (Shi 2010; Davies and Hazelton 2010; Davies et al. 2016; Tiwari and Rushton 2005). Hence, adjusting for *spatial* variation of the background is a common practice to date. However, adjusting for *spatial and temporal* variation of the background is a novelty, to the best of our knowledge. By omitting time in their models, existing methods ignore temporal dynamics (temporal variation) of the background, and are therefore unable to capture rapid population change.

However, we currently find ourselves in the age of migration (Castles et al. 2013), where individuals move from their residential location for many reasons: forced migration due to climate change (Martin 2001), conflicts (Mitchell 2011), or to find labor (Münz 2007). For instance, cities experience waves of urbanization (Meentemeyer et al. 2013), suburbanization (Lang and Simmons 2003), re-urbanization and counter-urbanization (Champion 2001). Hence, it is no longer acceptable to ignore temporal population dynamics, which is especially relevant for longitudinal studies. In addition, population data are becoming available at finer spatial and temporal resolutions, and given current technological advances, it is foreseeable that this development will continue (Bhaduri et al. 2007; Huang et al. 2020; Kang et al. 2020). This calls for an extension of the current kernel methods for computing disease risk to address background populations that change over space *and* time.

Adaptive kernels allow for variation in bandwidth (a.k.a search radius) across the study area, while fixed-bandwidth kernels do not. Therefore, fixed-bandwidth kernels may not properly capture human dynamics at varying geographic scales (Yuan 2018). They tend to oversmooth, leading to a loss of spatial detail (Goodchild 2001) and conceal regions of interest (Shi 2010), especially when analyzing large-scale human geographic phenomena, where events like disease cases emerge out of the background. While fixed-bandwidth kernels establish constant areal support, a kernel that adapts its bandwidth to the background is useful to establish constant population support (Carlos et al. 2010). This allows for comparison of disease rates across regions, which is especially suited for analyzing chronic diseases, such as lung cancer (Shi 2010). On the other hand, a kernel that adapts to surrounding cases sacrifices the constant population support property and establishes constant case support. This is suited for analyzing communicable disease, as distance between cases is a reasonable approximation of interaction between infected individuals, a common cause of disease spread (Bhopal 2016). Hence, adaptive kernels allow for measuring the scale over which a point process operates (Fotheringham et al. 2017; Xu et al. 2017).

**Fig. 1:**
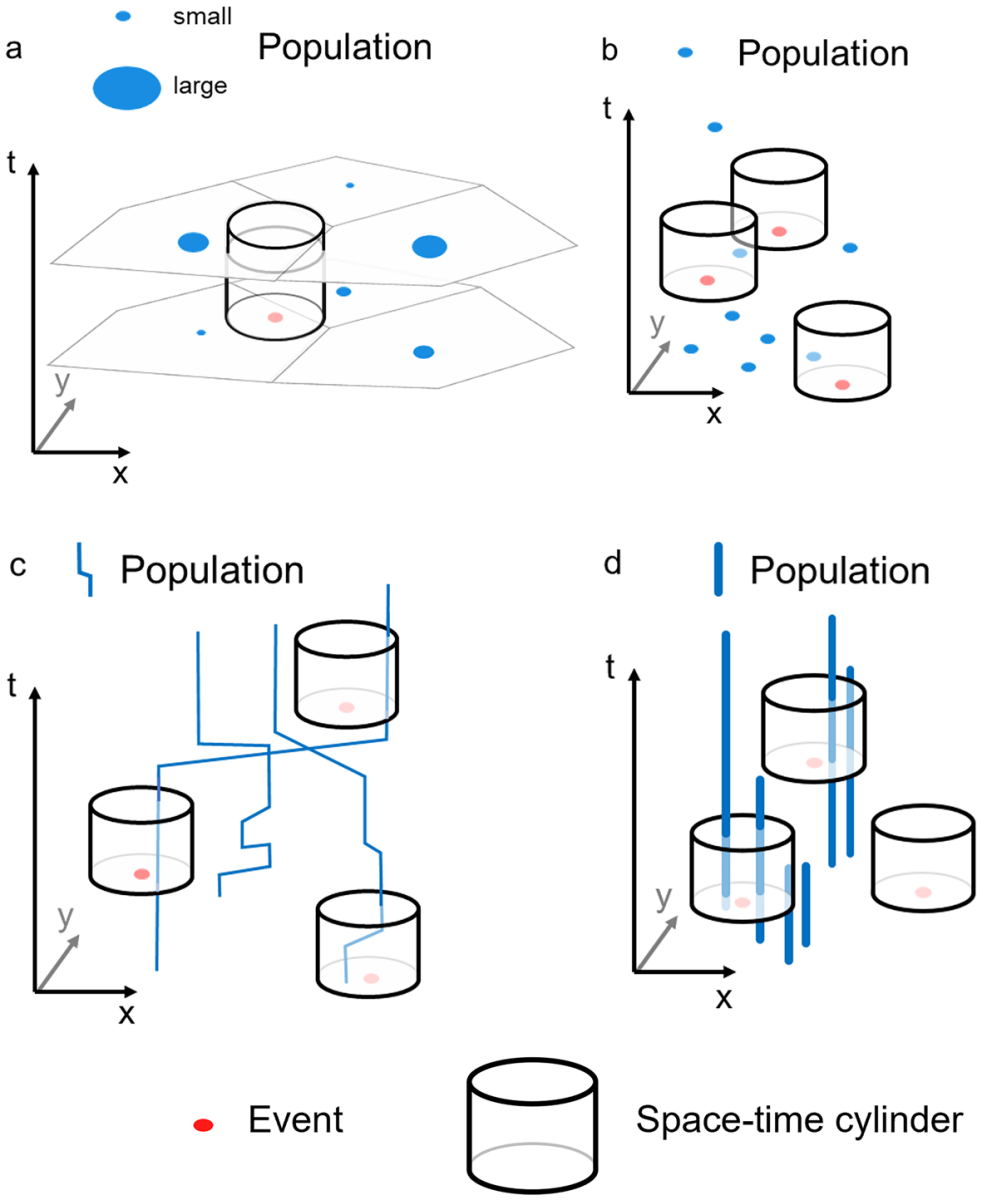
Different representations of the background: **a** centroids (e.g. census tracts), **b** social media posts (e.g. tweets), **c** trajectories of individuals, **d** residential locations

It is important to recognize that there are many ways to obtain a representation of the background (Fig. 1), i.e. from population data at high spatial and temporal resolution. Apart from census data (Fig. 1a), scientists have used social media posts such as tweets (Fig. 1b) as a proxy for population (Malleson and Andresen 2015b). Alternatively, trajectories of individuals (Fig. 1c) may be created through retrospective activity diaries (Chen et al. 2011; Kwan 2000, 2004), or migration history datasets (Shaw et al. 2008). Lastly, residential location may be used to establish the population at risk (Fig. 1d). In addition to the spatial coordinates *x* and *y*, the temporal coordinates *t*_*1*_ and *t*_*2*_ may be known, which represent start and end date of residence. In summary, the representations of population in Fig. 1 are profoundly different from each other. Besides availability, the following principle should guide the choice of population data: The scale of the population data should match the scale of the case data. For instance, if we use patient residential locations, we may want to use control data, as activity diaries population information would be too detailed. As the background can be represented in different ways, adaptations to the kernel density method for computing risk estimates are necessary to accommodate such diversity.

In this study, we address spatially and temporally varying backgrounds in kernel density estimation, a key omission of many existing applications. We introduce ST-DB, a space–time kernel density estimator that considers a **s**patially *and* temporally dynamic background. We compare ST-DB with its purely spatial counterpart and address the question of whether adding time to our analysis yields different estimates of disease risk. While our study focuses on introducing ST-DB and its application to spatiotemporal analysis of infectious disease, it can be applied to any geographic phenomenon that involves point events that emerge out of a background. This article is organized as follows: We explain our methodology, as well as our dataset in Sects. 2–4, illustrate our results in Sect. 5, and finally present discussion and conclusions in Sects. 6 and 7.

## Methods

### Kernel density estimation for static backgrounds

Kernel density estimation (KDE) is an essential method for analyzing spatial point (a.k.a. ‘event’) patterns (Silverman 1986). It results in a smooth surface of density estimates by imposing a regular grid of points (‘pixels’) on the study area. The density for each grid point is computed based on neighboring events, as follows: The kernel, a circular window with radius *h*_s_ (‘bandwidth’, *s* denotes ‘spatial’) is centered on a data point. Any grid point located within the kernel receives a contribution (a.k.a. ‘weight’) toward its density estimate. The contribution is determined by their distance to the data point in the center, which is plugged into the kernel function (*k*_s_, closer proximity results in higher contribution). Lastly, the weights are summed for each grid point, as multiple data points can contribute to a given grid point. We repeat the procedure for each data point and hence, create a density surface based on the observed point data. For a given grid point *(x, y)*, kernel density $\hat{f}\left( {x,y} \right)$ is calculated as follows (Eq. 1):

1 $$\hat{f}\left( {x,y} \right) = \frac{1}{{nh_{{\text{s}}}^{2} }} \mathop \sum \limits_{i = 1}^{n} k_{{\text{s}}} \left( {\frac{{d_{{i\left( {x,y} \right)}} }}{{h_{{\text{s}}} }}} \right)$$

where *n* is the number of data points within the study area, *k*_s_ is the kernel function, and *d*_*i*_(*x*,*y*) is the distance between the grid point and data point *i*. Kernel functions like Epanechnikov, Gaussian, or Biweight are widely used and accepted (Bowman and Azzalini 1997). We use the Epanechnikov kernel function (Epanechnikov 1969) in all of our analyses due to its popularity for spatial- and spatiotemporal analysis.

### Kernel density estimation for dynamic backgrounds

For many geographical research questions, density as the distance-weighted number of points per *unit area* may not provide a suitable answer (Bithell 1990). In the case of estimating disease risk, mapping the distance-weighted number of disease cases per *unit population-at-risk* rather than per *unit area* might be more realistic. The latter assumes that geographic distance is the sole determinant of the contribution of a case to the disease risk at a grid point (Shi 2010). As a result, an area of elevated risk identified by KDE might merely reflect a large local background population (Bithell 2000). Depending on the phenomenon under study, the population-at-risk can exhibit an uneven distribution in space and time, include all or only certain segments of the population (i.e. for COVID-19, elderly individuals and people with comorbidities are at increased mortality risk), and may be a sample of the full population-at-risk. This is referred to as the background population, or simply ‘the background’ (Carlos et al. 2010). A generic method to deal with a spatially varying background is to compute the risk $\left( {\hat{r}} \right)$ at location (*x, y*) by dividing the density of cases (*c*) by the density of the background population (*p*), shown in Eq. 2 (Davies and Hazelton 2010).

2 $$\hat{r}\left( {x,y} \right) = \frac{c}{p}$$

We use Eq. (2) to compute the risk at any location (*x, y*) by centering the kernel on each data point *i* (disease case). We then compute *i*’s contribution to risk at surrounding grid points by factoring in the population within the kernel. Therefore, the contribution of *i* to the risk at (*x, y*) is determined by the population near *i*, and not by the population near (*x, y*). In other words, the contribution of a case to the disease risk at a particular grid point is controlled by the local population surrounding that case, rather than the population surrounding the grid point. This distinction is relevant as it can result in different risk estimates under spatially varying backgrounds (Shi 2010).

**Fig. 2:**
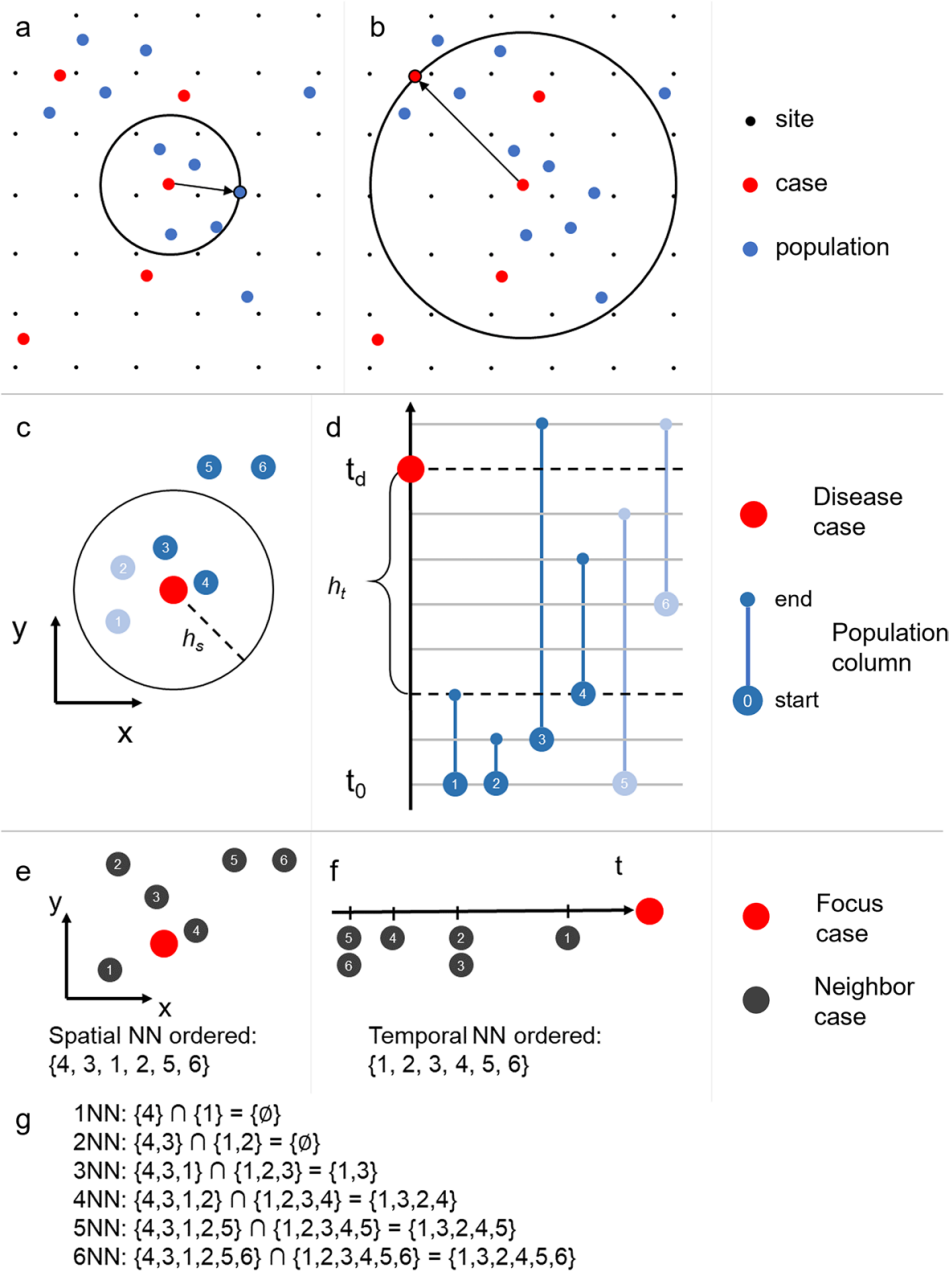
Top: adaptive bandwidth kernel with spatially varying background, **a** kernel adapts to population, **b** kernel adapts to cases. Note that *support* in **a** is 5 people and in **b** is 3 cases (the case in the center of the circle is not counted toward the *support* threshold). Middle: Quantifying the population within the kernel, **c** spatial view, **d** temporal view. In this example, the population within the kernel is 8 people-days. Only population columns 3 and 4 are inside the kernel spatially and temporally and the sum of their lengths inside the kernel is 5 + 3 = 8. Bottom: spatiotemporal nearest neighbors (NN), **e** spatial NN, **f** temporal NN, **g** intersection of spatial and temporal NN

Fixed kernels have constant bandwidth (search radius), whereas adaptive kernels allow the bandwidth to adapt to local conditions (Sain 2002; Brunsdon 1995). In our case, the kernel can either adapt to the background (Fig. 2a) or neighboring cases (Fig. 2b). While a fixed kernel results in constant areal support for each case, an adaptive kernel establishes either a constant population or case support. Areas that exhibit a high density of disease events (‘clusters’), for example, are often sought out for prevention efforts (Coleman et al. 2009). As infectious diseases spread between individuals in close proximity (Salathé et al. 2010), an area where cases cluster may be characterized as ‘high risk,’ whereas an area where cases are sparse may be ‘low risk’ (Bhopal 2016; Riley 2007). Therefore, we choose a kernel that adapts to neighboring disease cases and adjusts the estimate to the background within.

We achieve this by centering the kernel on a disease case and start increasing the bandwidth until it encircles a specified number of neighboring cases (the *support* threshold). Note that as the kernel expands, the case in its center will expand the spatial range of its contribution to disease risk. In other words, as the circle grows outward, seeking support, more grid points will receive contribution from the disease case in its center.

Shi (2010) proposes an adaptive bandwidth kernel density estimator (Eq. 3), which corresponds to Fig. 2a):

3 $$\hat{r}\left( {x,y} \right) = \mathop \sum \limits_{i = 1}^{n} k_{{\text{s}}} \left( {\frac{{d_{{i, \left( {x,y} \right)}} }}{{h_{{\text{s}}} \left[ {p\left( {x_{i} ,y_{i} } \right)} \right]}}} \right)$$

where the bandwidth *h*_s_ is a function of the local population density *p* at the location (*x*_*i*_, *y*_*i*_) of case *i*. The weight of *i* for (*x*, *y*) is divided by the population in the kernel. This method results in disease risk values that are defensible in health studies, while also being more statistically comparable (Carlos et al. 2010; Shi and Wang 2015; Shi 2010).

Equation (4) denotes S-DB, the purely spatial kernel density estimator for dynamic backgrounds that adapts to the neighboring cases, which corresponds to Fig. 2b):

4 $$\hat{r}_{{{\text{S}} - {\text{DB}}}} \left( {x,y} \right) = \mathop \sum \limits_{i = 1}^{n} k_{{\text{s}}} \left( {\frac{{d_{{i, \left( {x,y} \right)}} }}{{h_{{\text{s}}} \left[ {c\left( {x_{i} ,y_{i} } \right)} \right]}}} \right)$$

Here the bandwidth *h*_s_ is a function of the local case density *c* at the location (*x*_*i*_, *y*_*i*_) of case *i*. The density contribution of *i* to the locations within bandwidth is then divided by the population in the kernel. Note that both estimators (Eqs. 3, 4) require choosing the *support* threshold value. We circumvent this choice by analyzing the sensitivity of the resulting risk estimates to the *support* threshold value.

### Space–time kernel density estimation for static backgrounds

So far, we have ignored the temporal dimension in the discussion. Many geographic studies do not consider the temporal dimension or employ time-flattening: collapsing the temporal dimension into a single 2D map, which represents the entire study period (Bach et al. 2016). Another approach discretizes time into a number of time slices, which can be displayed as small multiples (Boyandin et al. 2012). However, both methods are limited in their ability to depict patterns of spatiotemporal point events: Time-flattening ignores any temporal variation in the data, and the small multiples approach is not scalable (Delmelle et al. 2014).

Space–time kernel density estimation (STKDE) extends traditional bivariate KDE with the temporal dimension and is suited for characterizing spatiotemporal patterns of spatial point events with a timestamp (Nakaya and Yano 2010). STKDE produces density estimates for a spatiotemporal grid of points (‘voxels’) based on the proximity and number of surrounding point data (Delmelle et al. 2014; Brunsdon et al. 2007). We can visualize the density estimates within a space–time cube (Hagerstrand 1970; Bach et al. 2016; Hohl et al. 2016; Desjardins et al. 2019; Gao 2015; Nakaya and Yano 2010; Demšar et al. 2015) that has two spatial (*x*, *y*) and a temporal dimension (*t*). STKDE is computed as follows: We center the bottom of the kernel, a cylindrical window defined by its circular base with radius *h*_s_ (spatial bandwidth) and height *h*_t_ (temporal bandwidth) on a data point. Any voxel located within the kernel receives a contribution (or weight) toward its density estimate, as a case imposes risk only to the time-period after the event. The contribution is determined by the distance between voxel and data point in the center, which is plugged into the spatial and temporal kernel functions (*k*_s_, *k*_t_). Lastly, the weights are summed for each voxel, as multiple data points can contribute to a given voxel. We repeat the procedure for each data point and hence, create a density volume based on the observed point data. For a given voxel (*x*, *y*, *t*), density is calculated as follows (Eq. 5):

5 $$\hat{f}\left( {x,y,t} \right) = \frac{1}{{nh_{{\text{s}}}^{2} h_{{\text{t}}} }} \mathop \sum \limits_{i} k_{{\text{s}}} \left( {\frac{{d_{{i,\left( {x,y} \right)}} }}{{h_{{\text{s}}} }}} \right)k_{t} \left( {\frac{{d_{i,\left( t \right)} }}{{h_{{\text{t}}} }}} \right)$$

Every voxel *s* with coordinates (*x*, *y*, *t*) receives a density estimate $\hat{f}\left( {x,y,t} \right)$, which is determined by distance and number of neighboring data points *i*. Data points in the neighborhood of *s* are weighted by the spatial and temporal kernel functions, *k*_s_ and *k*_t_, which are computed as separate components, and then multiplied to calculate the contribution of a given data point to the density estimates on surrounding grid points. Lastly, *d*_*i,*(*x*,*y*)_ and *d*_*i*,(*t*)_ are the spatial and temporal distances between voxel and data point, respectively.

### Space–time kernel density estimation for dynamic backgrounds

Here, we discuss the extension of STKDE to account for spatially and temporally varying backgrounds (Eq. 6). It is the temporal extension of Shi’s case-side adaptive bandwidth kernel density estimator:

6 $$\hat{r}\left( {x,y,t} \right) = \mathop \sum \limits_{i} k_{{\text{s}}} \left( {\frac{{d_{{i,\left( {x,y} \right)}} }}{{h_{{\text{s}}} \left[ {p\left( {x_{i} ,y_{i} } \right)} \right]}}} \right)k_{{\text{t}}} \left( {\frac{{d_{i,\left( t \right)} }}{{h_{{\text{t}}} \left[ {p\left( {t_{i} } \right)} \right]}}} \right)$$

The spatial- and temporal bandwidths *h*_s_ and *h*_t_, respectively, are a function of the local population density *p*(*x*_*i*_, *y*_*i*_), *p*(*t*_*i*_) at space–time location (*x*_*i*_, *y*_*i*_, *t*_*i*_) of data point *i*. The background is assessed within a half cylinder moving through 3D space, which means that we consider the population within the kernel until the disease case occurs, but not after. A kernel that adapts to the background population is useful to establish constant population support (constant *p* in Eq. 2), rather than constant areal support, which is the case with fixed bandwidth kernels.

As seen in Sect. 2.2, it may make sense to adapt the bandwidth to the surrounding cases *c*(*x*_*i*_, *y*_*i*_, *t*_*i*_) instead of the local population *p*(*x*_*i*_, *y*_*i*_, *t*_*i*_) (Eq. 7). Therefore, we define the kernel density estimator for spatially and temporally dynamic backgrounds (ST-DB) as follows:

7 $$\hat{r}_{{{\text{ST}} - {\text{DB}}}} \left( {x,y,t} \right) = \mathop \sum \limits_{i} k_{{\text{s}}} \left( {\frac{{d_{{i,\left( {x,y} \right)}} }}{{h_{{\text{s}}} \left[ {c\left( {x_{i} ,y_{i} } \right)} \right]}}} \right)k_{{\text{t}}} \left( {\frac{{d_{i,\left( t \right)} }}{{h_{{\text{t}}} \left[ {c\left( {t_{i} } \right)} \right]}}} \right)$$

Here, the spatial and temporal bandwidths *h*_s_ and *h*_t_ expand until a specified number of neighboring disease cases is found within the cylindrical kernel. The density contribution of the disease case *i* to the voxels within bandwidth is then divided by the population in the kernel. As population information might be available in different formats and conceptualizations (see Sect. 1), we pick the population columns model (Fig. 1d) to illustrate the utility of our approach. The within-kernel population is computed by summation of the segment length of all population columns within the cylinder (Fig. 2c, d). The sum represents the number of individuals and their length of exposure to the disease case. It is measured in people-days (an analogy to the term ‘man-hours’ used to quantify the amount of work that can be done by one person within this period).

The *support* can be achieved in multiple ways. In search for neighbors, we could either exclusively expand the spatial bandwidth, or exclusively the temporal bandwidth, or both in an alternating pattern. Therefore, ambiguity arises by the choice of search strategy. To solve this problem, we need to unify the spatial and temporal dimensions, allowing us to expand the bandwidths simultaneously. We employ the *k*-nearest neighbors (kNN) method (Jacquez 1996) for this task as follows:

1. Generate two ordered sets for each disease case: (1) the spatial *k*-nearest neighbors (Fig. 2e) and (2) the temporal *k*-nearest neighbors (Fig. 2f) of case *i*.
2. Compute the cardinality *card()* of the intersection between the two sets (Fig. 2g).

Starting with *k* = 1, we increase *k* and apply the procedure until *card()* equals the *support* threshold. We then compute the spatial and temporal bandwidths *h*_s_, *h*_t_, respectively, as the spatial and temporal distance of the farthest point in the intersection set to the case. Using this procedure, we unify the spatial and temporal dimensions, enabling search for the support in adaptive-bandwidth kernel density estimation for spatially and temporally dynamic backgrounds. Therefore, we solve the multiway problem for ST-DB.

## Data

### Case data

**Fig. 3:**
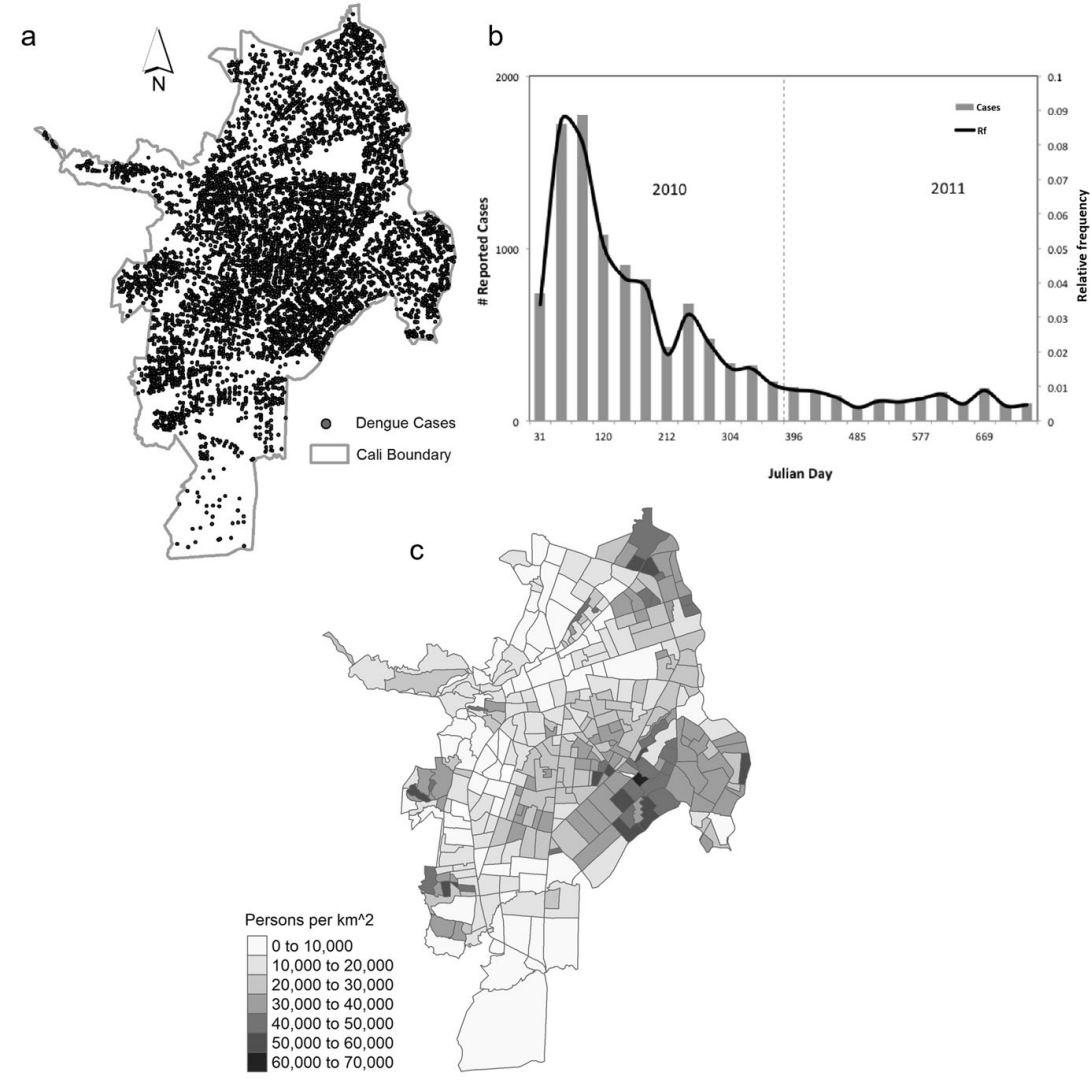
The dengue fever dataset: **a** spatial and **b** temporal distributions. **c** Population density of Cali, Colombia in 2010

For the case study we use dengue virus data from the city of Santiago de Cali, Colombia (‘Cali’). Cali is located in the southwest of Colombia, which exhibits very suitable conditions for the Aedes Aegypti mosquito (the principal mosquito vector for dengue virus). The city of Cali has a population of around 2.5 million and it is considered an endemic area for dengue fever (Cali 2010). The dengue fever dataset includes individual case records, for which *x*- and *y*-coordinates of the patient residential address, as well as the time of diagnosis are available (Delmelle et al. 2014). A total of 11,056 cases were observed in our 2010–2011 study period. Figure 3 presents the spatial distribution of cases within the city (a) and the temporal distribution through the two years of study (b).

### Population Data

Population data are obtained from the Administrative Planning Department from the city of Cali (Cali 2019), which include population projections by neighborhood from 2006 to 2036. We eliminated six neighborhoods that had zero population (parks, sports complexes, military facilities), which resulted in a total of 334 neighborhoods in our study area. We computed summary statistics for dengue fever case counts and population at the neighborhood level, as well as their change, for the years 2010 and 2011 (Table 1) and mapped the 2010 population density by neighborhood (Fig. 3c). Peripheral areas, especially to the east of the city, have a high concentration of population, while neighborhoods in the central part of the city which constitute an extension of the city core have lower population density levels.

**Table 1:** Summary statistics of neighborhood-level dengue fever case counts and population 2010–2011

	Dengue fever cases		Population	
	2010	2011	2010	2011
Min	0	0	59	58
Max	143	34	49,978	50,581
Mean	28.4	4.6	6,556.9	6,629.3
Standard deviation	25.3	4.4	6,369.2	6,478.8

**Fig. 4:**
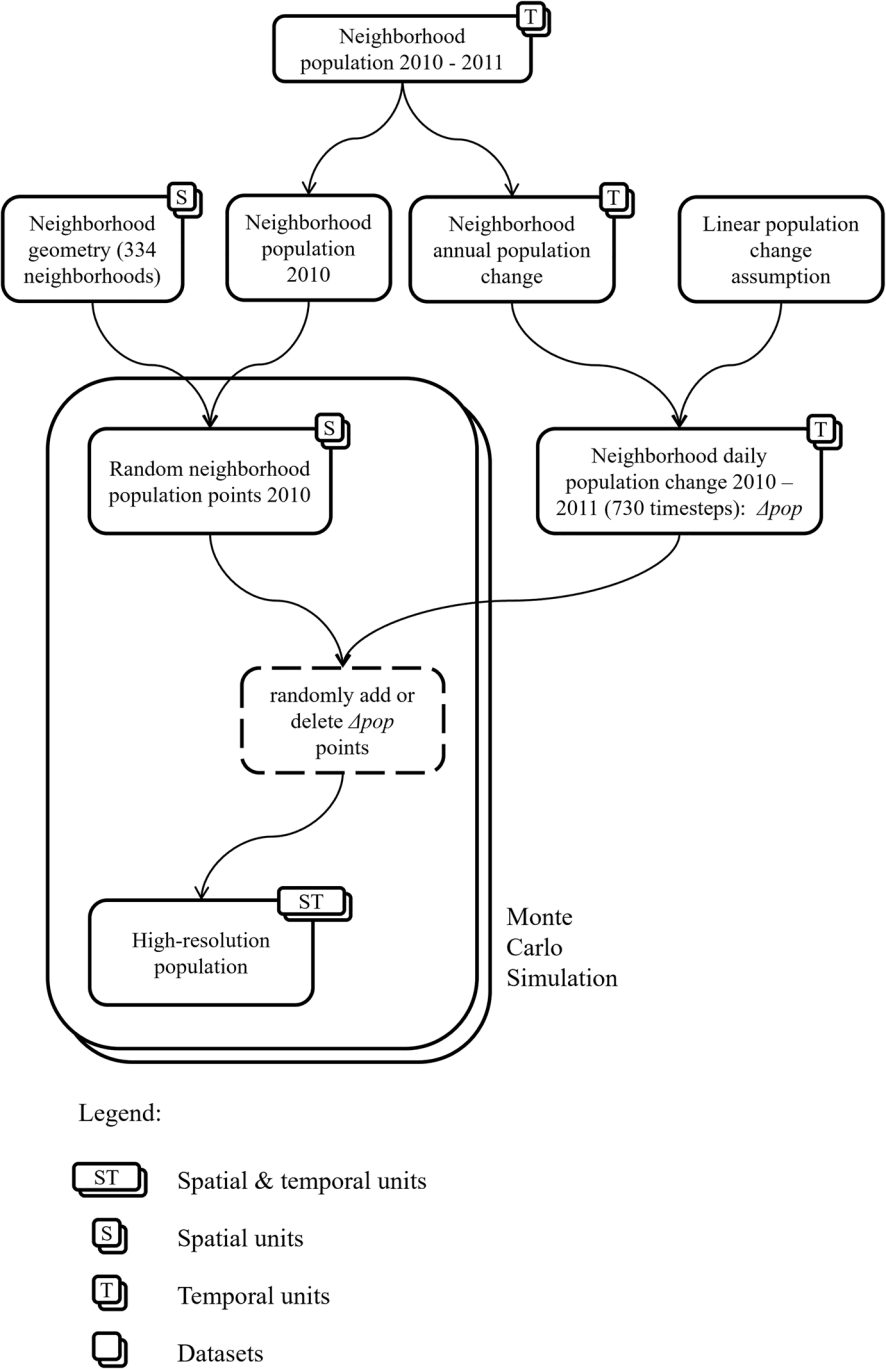
Flowchart for population disaggregation

We put forward a simple procedure to disaggregate population data from their spatial and temporal units (neighborhoods, years) to individual level (Fig. 4), similar to Jacquez and Jacquez (1999), Shi (2009) and Luo et al. (2010):

1. We distribute the population of the first year (2010, ‘the initial population’) within each of the 334 neighborhoods at random locations, which are noted in our population table as [*x, y, t*_1_*, t*_2_]-tuple (*x*-coordinate, *y*-coordinate, first day of residence, and last day of residence). For instance, a person who lived in Cali from 1/1/2010 to 31/12/2011 would have *t*_1_ = 1 and *t*_2_ = 730. Every neighborhood receives a number of [*x, y, t*_1_*, t*_2_]-tuples commensurate with its total population of 2010.
2. Using the yearly neighborhood population counts 2010–2011, we compute annual population change (increase/decrease) for each neighborhood. We scale down the annual change to daily values, assuming linear change. Hence, we compute the daily change in population by dividing the annual change by 365.
3. For each day within 2010–2011 (which amounts to 730 timesteps), we add random points commensurate with the population increase and set their *t*_1_ to the current day. In case of population decline, we randomly pick existing points commensurate with the population decline and set their *t*_2_ to the current day.

Hence, we create a spatiotemporal, individual-level population dataset, equivalent to the population ‘columns’ in Fig. 1d.

## Analysis

**Fig. 5:**
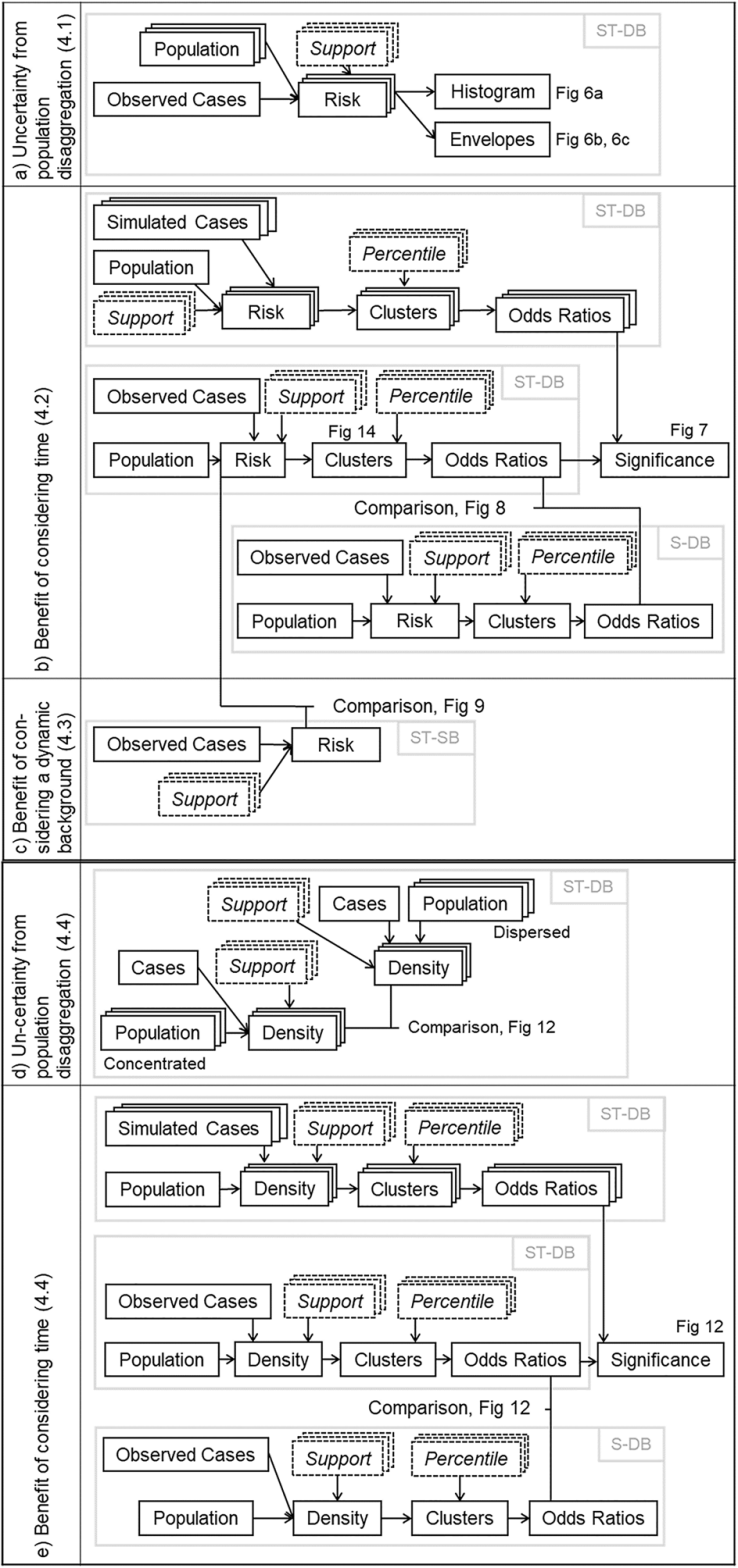
Unified methodological framework. Case study (**a**–**c**), simulation study (**d**, **e**). Black boxes denote data items while arrows in between denote processes on the data items; multiple overlapping boxes denote multiple simulated datasets; dashed line boxes are parameters; gray boxes are methods

We conduct analyses within our methodological framework (Fig. 5) that summarizes our analytical steps. It also contains references to sections in the text where the corresponding steps are detailed, as well as to figures that show the results.

### Uncertainty from population disaggregation

We quantify uncertainty from population data disaggregation (Sect. 3.2), as the process includes random elements. Therefore, we create 99 disaggregated population datasets, leaving the disease data unchanged, and compute 99 grids of risk estimates, which allows us to extract upper and lower envelopes as the maximum and minimum value for each grid point (Fig. 5a). This results in variance of risk, which is a measure of the uncertainty resulting from the random elements involved in disaggregating population data. To quantify the uncertainty, (1) we compute a histogram of the differences between the upper and lower envelope, and (2) visualize them within the space–time cube. If the histogram indicates that the difference is mostly small, we conclude that uncertainty from population disaggregation is small. In addition, the depiction within the space–time cube enables for detecting patterns of where and when the results may be subject to high uncertainty.

### Benefit of considering time

In this study, we compare ST-DB (Eq. 7) with its purely spatial counterpart, S-DB (Eq. 4) and assess whether incorporating a temporally dynamic background improves the ability to detect areas/periods of high disease risk (a.k.a. ‘clusters’, Fig. 5b). We apply the following procedure using both approaches (ST-DB and S-DB):

- Step 1: We compute disease risk estimates.
- Step 2: We denote the grid points with *n*-th percentile of risk or higher as clusters (*percentile* threshold).
- Step 3: We compute the strength of the clustering using odds ratios (within-cluster risk vs. out-of-cluster risk).
- Step 4: We compute cluster significance using Monte Carlo simulation.

We run both methods with the same data but ignore the temporal dimension for S-DB (Step 1). After computing disease risk estimates (ST-DB produces a 3D grid, S-DB a 2D grid), we delineate disease clusters as follows (Step 2): We apply the *percentile* threshold and pick the grid points with the *n*th-percentile of risk or higher and label them as disease cluster. We measure cluster strength by computing odds ratios (Step 3), which is the ratio between risk inside and outside of the clusters (Bland and Altman 2000; Kulldorff 1997). A high odds ratio means that high-risk disease areas/periods have been delineated well from low-risk ones, as the ratio between cases and controls inside the cluster is much higher than outside. When comparing any two methods *A* and *B*, we say that method *A* delineates clusters better than method *B* if it produces a higher odds ratio. Lastly, we use Monte Carlo simulation to measure the statistical significance of clusters (Step 4). Method *A* is only better than *B* if it produces a higher odds ratio that is statistically significant. For each Monte Carlo simulation run, we randomize the locations of the observed disease cases by sampling from an inhomogeneous Poisson distribution with intensity that follows the population distribution of Cali, while using one realization of the population disaggregation procedure (as it turns out, uncertainty from population disaggregation is small). We compute odds ratios by applying Steps 1–3 for each simulation run, as well as for the observed dataset. The rank of the observed odds ratio among the simulated ones constitutes its *p*-value for testing the null hypothesis of Poisson distributed cases. We chose 99 simulation runs, which strikes a balance between computational feasibility and level of statistical confidence.^1^

To address the sensitivity of the resulting odds ratios to various parameter configurations, we apply Steps 1–4 for all combinations of different *percentile* threshold values for cluster delineation, and *support* threshold values for bandwidth selection (see Sect. 2.2). The *support* values are {5, 10, 15, 20, 25, 30, 35, 40, 45, 50, 55, 60, 65, 70, 75, 80, 85}, whereas the *percentile* values are {90, 91, 92, 93, 94, 95, 96, 97, 98, 99, 99.9, 99.99}. This allows us to draw odds ratio surfaces across different parameter configurations, and to compute the difference between ST-DB and S-DB. With the goal of illustrating the utility of our approach, we perform significance testing for all parameter configurations (ST-DB only).

### Benefit of considering a dynamic background

We illustrate the utility of adjusting kernel density estimates to a dynamic background by comparing ST-DB with space–time kernel density estimation for static background (ST-SB), where the background is ignored or assumed to be distributed homogeneously in space and time (Fig. 5c). We visualize density estimates from ST-DB and ST-SB within the space–time cube and expect high density areas/times to differ between the methods: while ST-SB allows for identifying clusters of high case density, ST-DB allows for identifying clusters of high disease risk.

### Simulation study

As Cali had little change in population during our study period, we conduct a simulation study in addition to our case study in Cali, Colombia, to compare ST-DB and S-DB (Fig. 5). In our simulation study, we implement a hypothetical 10% population growth within two scenarios. Each scenario consists of one realization of a random process, where we distribute the 2010 population (initial population) within the neighborhoods of Cali, but add the additional population (population increase) in different ways: (1) dispersed population increase following the existing population distribution, (2) concentrated population increase, where we distribute the population increase within a circle of radius of 3.5 km in the southern part of the city. The temporal signature of the population increase is a homogeneous Poisson process with *λ* = 2 for both scenarios. The results from the simulation study illustrate the benefits of ST-DB under a high population increase scenario. To reduce the computational cost, we use a random subset (*N* = 550) of the original case data in our simulation study. As with our case study, we assess the uncertainty from population disaggregation (Fig. 5d), as well as the benefit of considering time (Fig. 5e), while using the following parameter configurations: support = {9, 12, 15, 18, 21}, and percentile = {90, 91, 92, 93, 94, 95, 96, 97, 98, 99, 99.9, 99.99}.

## Results

### Uncertainty from population disaggregation

**Fig. 6:**
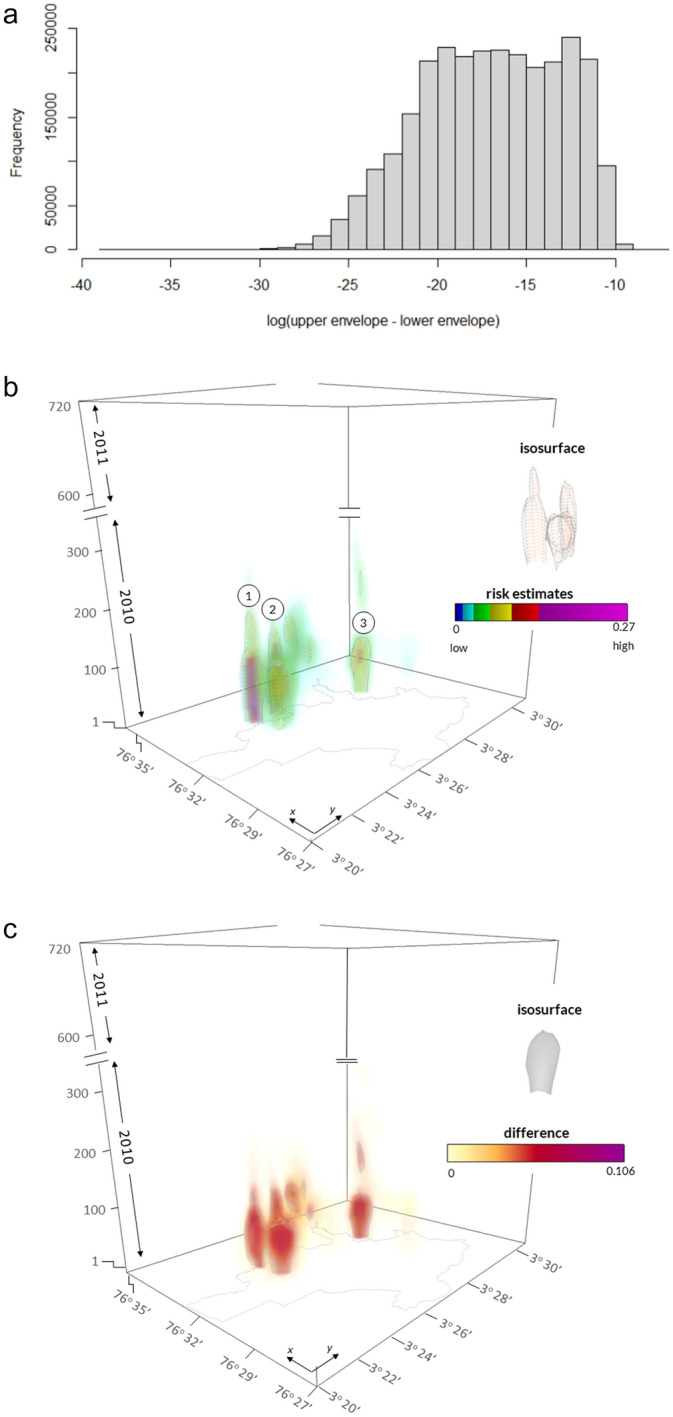
**a** Histogram of differences between upper and lower envelope (support = 5); **b** spatiotemporal distribution of the upper simulation envelope (uncertainty from population simulation); **c** difference between upper and lower simulation envelope

In our case study, using the observed population change and the entire dengue fever dataset, the 99 population simulations resulted in 99 risk estimates for each grid point. To illustrate the uncertainty from these simulations, we computed the difference between maximum and minimum risk value for each grid point (upper and lower envelope, support = 5) and plotted their frequency within a histogram (Fig. 6a). The range of differences is 0.0–0.00037, which is a very small deviation, considering a range of risk values within 0.0–0.27. Therefore, the uncertainty from population simulation is rather small.

We plotted the upper envelope within the space–time cube (Fig. 6b) to provide a spatiotemporal depiction of the risk estimates. The lower envelope is not distinguishable from the upper envelope when viewing the scene at full extent, because the differences are very small. We can clearly see the two clusters of increased disease risk within the southwestern part of the city (Fig. 6b, points 1 & 2), commensurate with findings of Delmelle et al. (2014) and Hohl et al. (2016). These clusters are active from the very beginning of the study period and remain so for the first quarter of the study period. We also see another risk zone within the more central part of the city (Fig. 6b, point 3) which exhibits elevated disease risk estimates for approximately the first half of the study period.

The spatiotemporal distribution of the difference between upper and lower envelopes shows higher values where risk estimates are high as well (Fig. 6c). Hence, the differences follow the distribution of risk estimates. This result is expected and confirms that the uncertainty from population simulation is relatively small while following the spatiotemporal distribution of risk.

### Benefit of considering time

**Fig. 7:**
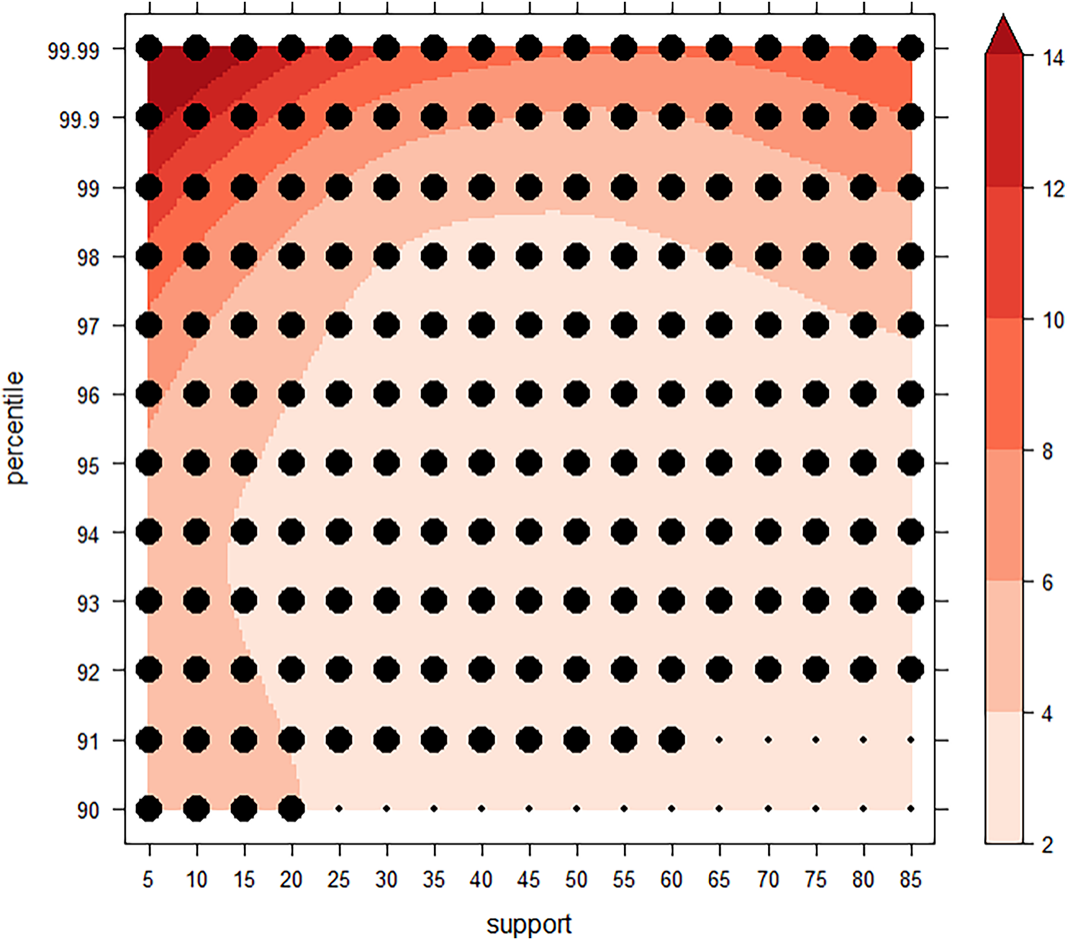
Difference between odds ratios S-DB—ST-DB. *Y*-axis: percentile threshold values. *X*-axis: support parameter values

Using the actual population of Cali during our study period (2010–2011), we compare S-DB and ST-DB and find that ST-DB performs better for the parameter space we assessed. Figure 7 indicates the difference between odds ratios produced by S-DB and ST-DB. The entire parameter space in Fig. 7 shows positive values, i.e. ST-DB has a higher odds ratio than S-DB. The differences range from 2.4 to 26.9 and increase toward higher *percentile* and lower *support* threshold values. Significance testing of clusters generated using ST-DB shows that all parameter combinations are significant except toward lower *percentile* and higher *support* values, i.e. percentile = 91 & support ∈ {65, 70, 75, 80, 85}, as well as percentile = 90 & support ∈ {25, 30, 35, 40, 45, 50, 55, 60, 65, 70, 75, 80, 85}.

**Fig. 8:**
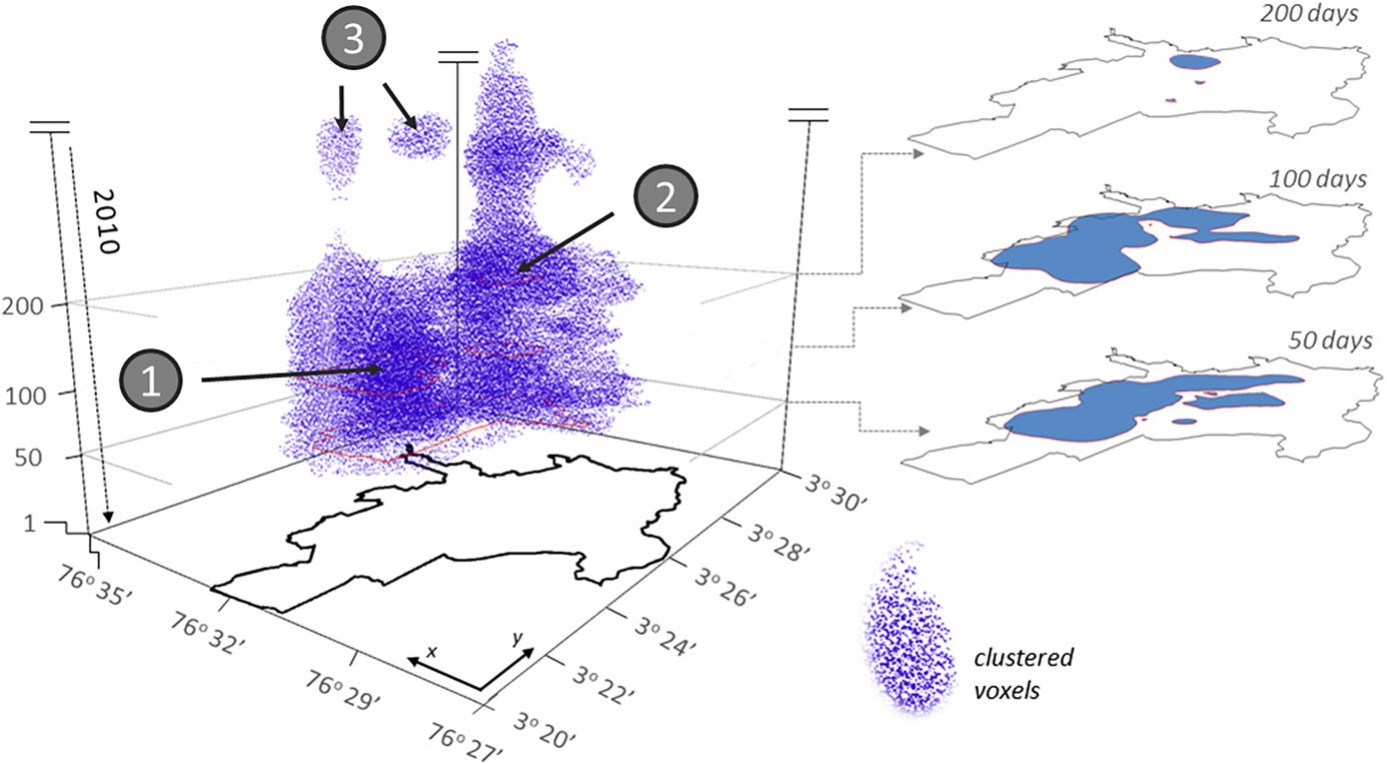
Voxels that form a significant cluster at the 0.01-level. Support = 45, percentile = 95

We found clusters of elevated dengue fever risk that are significant at the 0.01-level. Here, we illustrate an example using the parameter values of support = 45 and percentile = 95 (Fig. 8). The clustered voxels are distributed within the center of the city and within the first 314 days of the study period. The cluster has a large base at the beginning of the study period, which becomes thinner as time progresses. Therefore, distinct patterns of cluster shape are visible toward the upper end of the 314-day period. For instance, the cluster seems to consist of two parts: one in the South (Fig. 8, point 1) and in the North (Fig. 8, point 2). The Northern section of the cluster lasts substantially longer than its Southern counterpart. We are also able to make out detached ‘clouds’ of voxels that have been identified as clusters (Fig. 8, point 3). These ‘clouds’ indicate regions that experienced a resurgence of dengue risk after a period of little activity.

### Benefit of considering a dynamic background

**Fig. 9:**
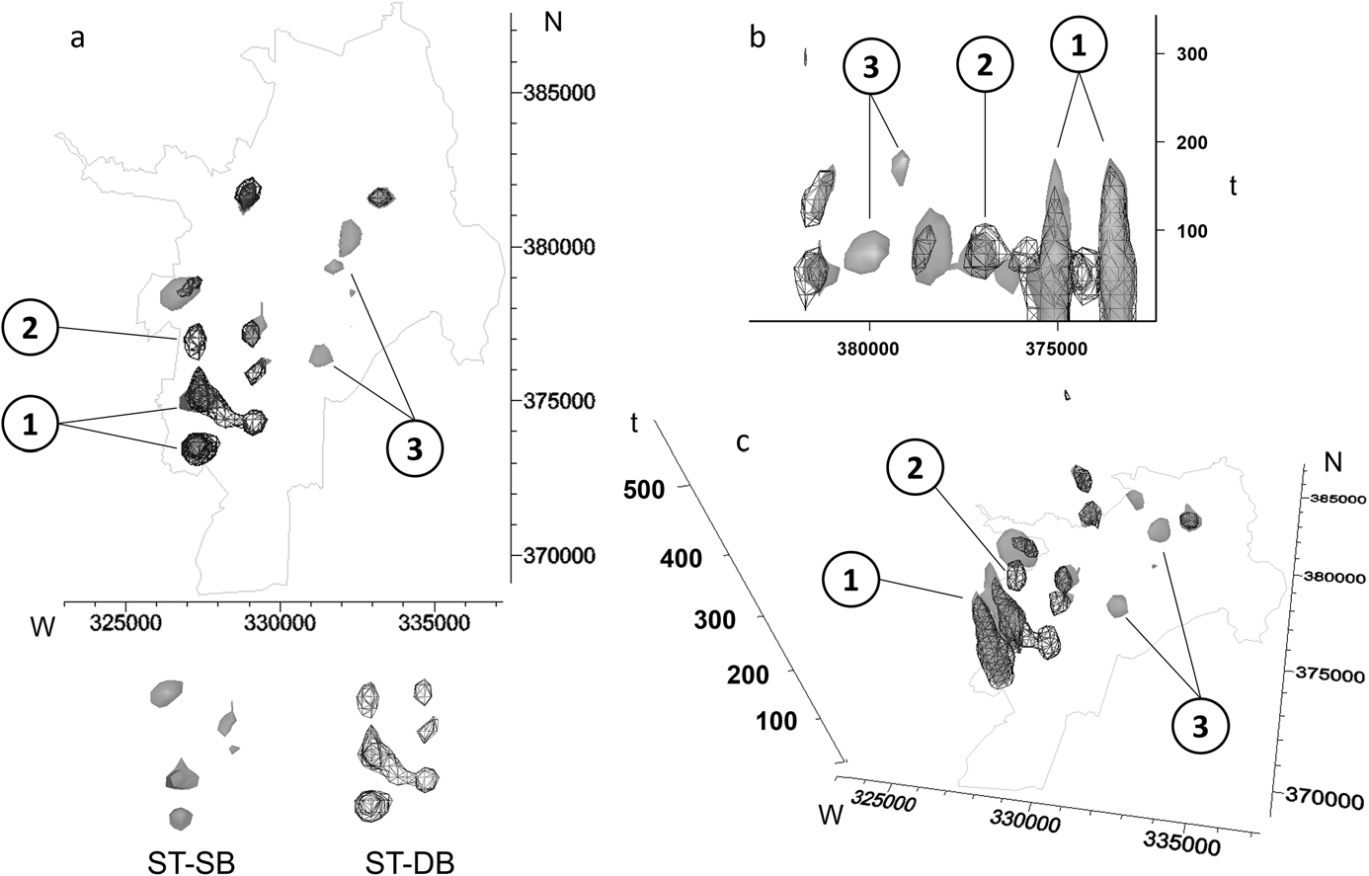
Difference between ST-DB and ST-SB

The density estimates produced by ST-SB exhibit an interesting pattern (Fig. 9). The isovalues are chosen to equalize the volume enclosed by the isosurfaces between the density grids of ST-DB and ST-SB. Figure 9 shows that there are three distinct areas: Point 1 has high values for both, ST-DB and ST-SB. This area corresponds to the well-known dengue clusters in the southwestern part of the city during the first 200 days of our study period (Delmelle et al. 2014). It means that these areas have high case density and a high population density, and therefore, conform to the expected spatiotemporal pattern of a disease epidemic. Point 2 is found just north of Point 1, where we observe high values for ST-DB, but not for ST-SB. It means we observe more cases than we would expect given the lower population density. Lastly, areas belonging to Point 3 are located in the central part of the city at around 50–200 days and exhibit high values for ST-SB, but not for ST-DB. They are on the fringe of the high population density areas in the eastern part of the city. As they are not visible when adjusting for the underlying population (ST-DB), these areas are within or below the expected pattern, yet using ST-SB alone, one would identify them as high-density areas.

### Simulation study

**Fig. 10:**
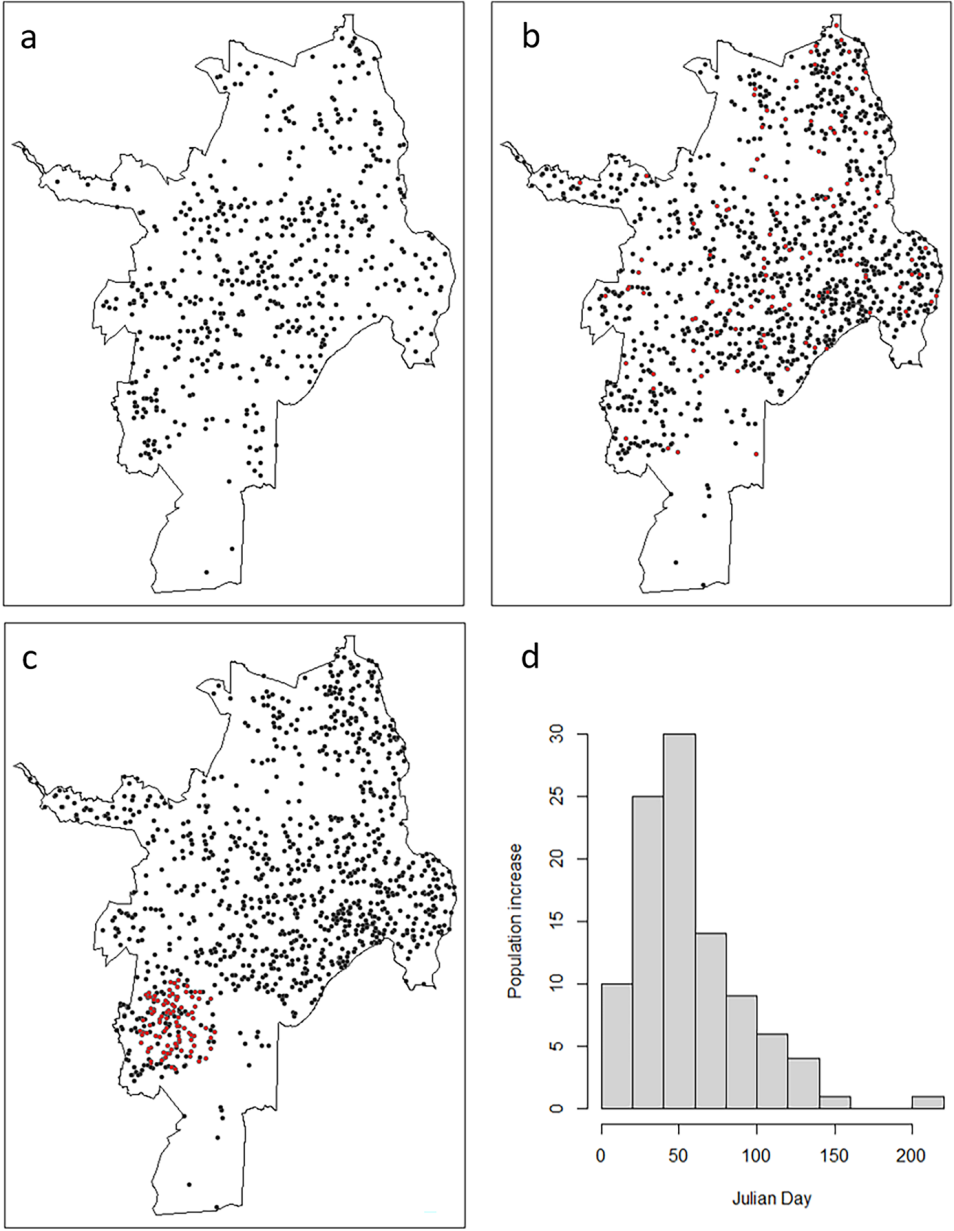
Simulation study—population simulation. **a** 550 cases (random sample from dengue fever dataset), **b** Simulated population dataset with initial population (black dots) and dispersed population increase (red dots), **c** Simulated population dataset with initial population (black dots) and concentrated population increase (red dots), **d** temporal signature of population increase, common to both population increase scenarios

The simulation study uses a random subset of the original dengue fever dataset of 550 points (Fig. 10a). We employ two scenarios that differ in the spatial pattern of population increase: dispersed (Fig. 10b), and concentrated (Fig. 10c). The temporal signature of population change is the same for both scenarios (Fig. 10d), exhibiting the strongest growth in the first half of the first year of our study period (2010).

**Fig. 11:**
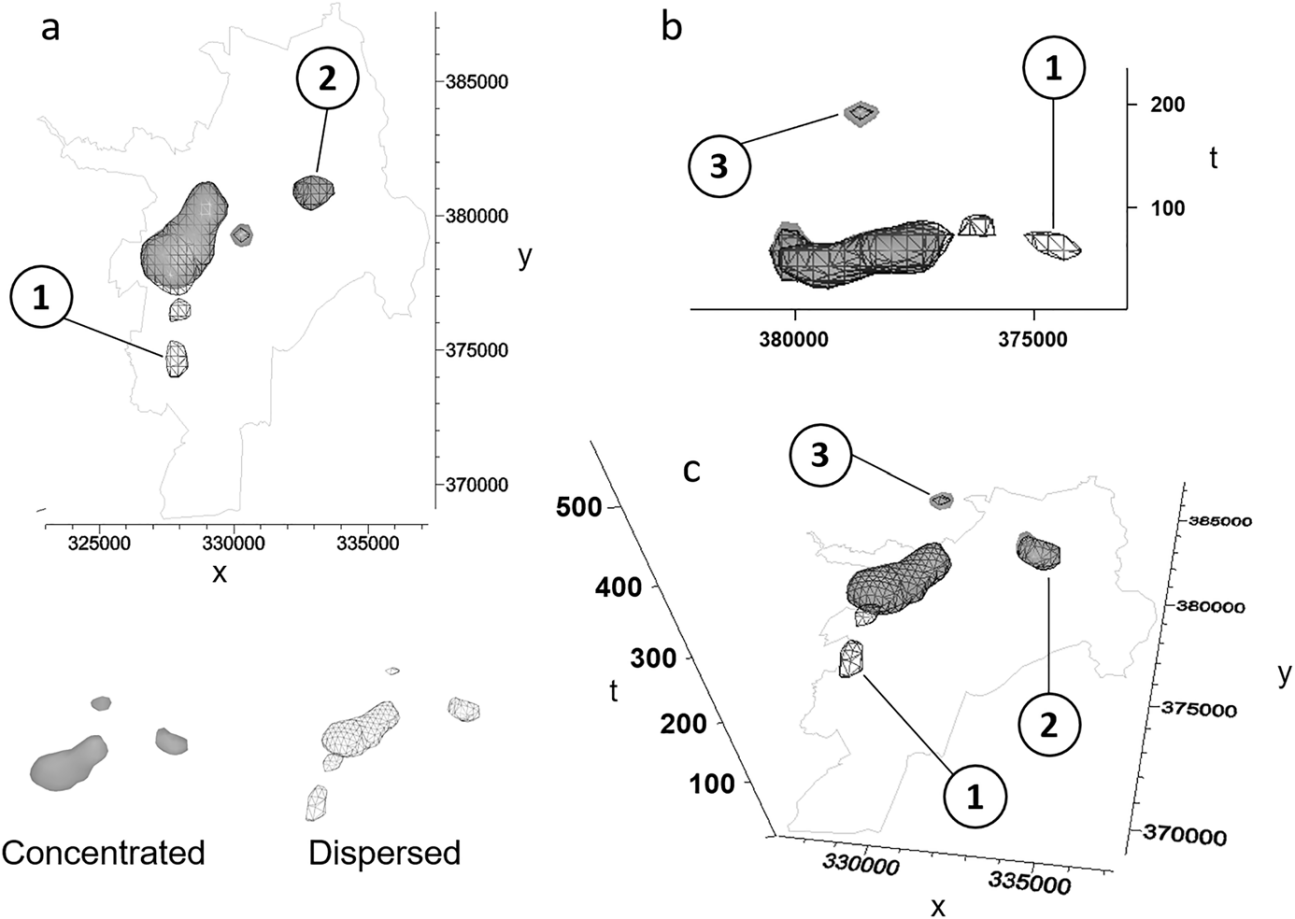
Risk estimates from ST-DB (support = 9). Isosurfaces of concentrated (gray color) and distributed population increase (mesh). View from **a** top, **b** West, **c** Southeast (elevated viewpoint). One simulation run out of 99 (population simulation)

It is apparent that the risk estimates of dengue fever cases resulting from ST-DB differ between the two simulation scenarios (Fig. 11). Figure 11 includes an isosurface for each scenario, where we chose isovalues in such a way that both isosurfaces enclose the same volume. This is different from choosing the same isovalue because the ranges of risk values differ. The concentrated population increase leads to lower risk estimates in the southwestern part of the city (see Fig. 11, Point 1) as compared to the estimates of the dispersed population increase scenario. This is evident from the isosurface of the dispersed scenario in Point 1, while the isosurface of the concentrated scenario is absent in that location. For both scenarios, the highest densities are found during the first 100 days of the study period, with a spatial concentration on the western parts of the city. Notable exceptions are two detached clusters in the central area of Cali, one emerging around day 100 (Fig. 11, point 2), one around day 200 (Fig. 11, point 3).

**Fig. 12:**
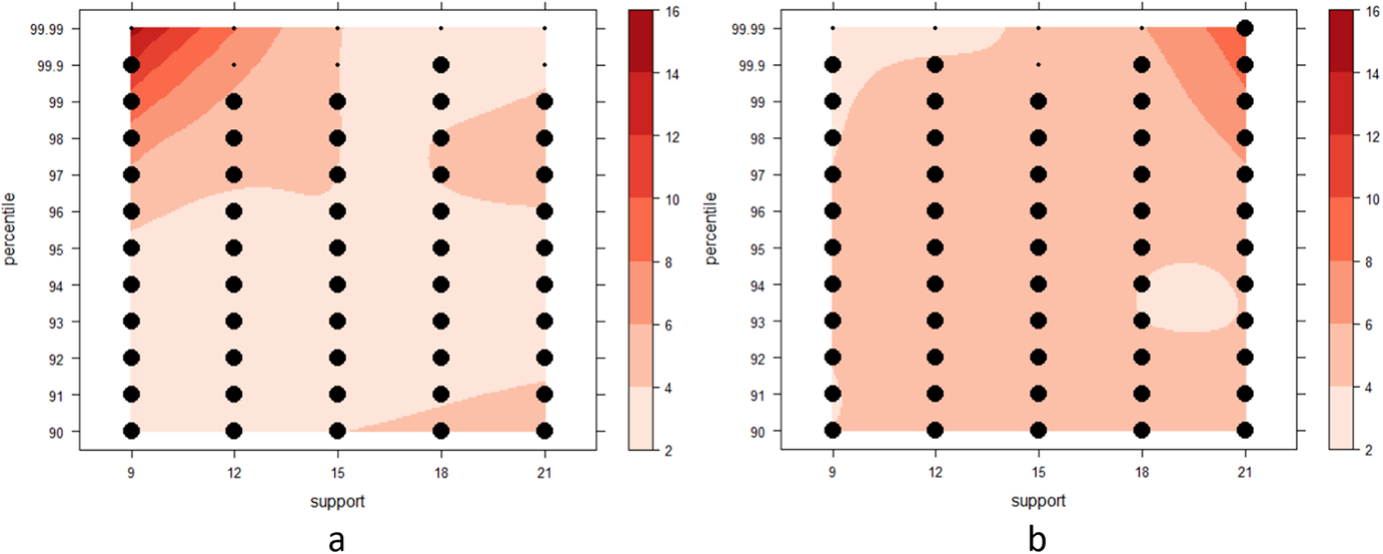
Simulation study—case simulation. Heatmap of difference between odds ratios of ST-DB and S-DB. Large dots indicate parameter configurations that resulted in significant clusters, small dots denote insignificant clusters. **a** Concentrated population increase, **b** dispersed population increase. *X*-axis: support parameter values. *Y*-axis: percentile threshold values

Comparing odds rations between ST-DB and S-DB, we find that ST-DB outperforms S-DB for the entire parameter space assessed (Fig. 12), as indicated by the positive difference all throughout. Significance testing shows that most clusters are significant, with few exceptions i.e. for extreme values of the *percentile* threshold parameter (percentile = 99.99).

## Discussion

Our experiments indicate that ST-DB yields higher odds ratios than S-DB for the parameter configurations assessed in both, our simulation and case studies (Figs. 7, 12). This is a positive result, suggesting a benefit of considering the temporal dimension in spatial analysis, as it may better allow us to capture the scale at which a point process operates. This implies that ST-DB, the key innovation of this study, improves our ability to delineate clusters of disease occurrence under spatially and temporally dynamic backgrounds. It is important to note that the ‘winner’ of the comparison between ST-DB and S-DB is not ‘better’ in a universal sense as it may merely result in different conclusions.

The choice of parameters is admittedly subjective, but we confirmed the validity of the resulting clusters by significance testing. Therefore, we created two measures of describing clusters: (1) We quantify the strength of a cluster by its odds ratio. The higher the ratio, the greater the difference in odds of contracting the disease inside vs. outside the cluster. (2) We quantify the significance of the cluster by its *p*-value. Therefore, the clustering of observed dengue cases by arbitrary parameter values generates higher odds ratios compared to all the Poisson distributed simulated datasets. The ability of choosing parameter values allows for ‘tuning’ the resulting significant clusters (Fig. 8). This way, authorities can choose the case support and risk threshold based on their needs and resources. If resources for disease prevention and mitigation are scarce, the risk threshold can be increased to produce smaller areas/periods of significant clusters.

We clearly saw that uncertainty from population disaggregation was low in our case study (Fig. 6a, c). This result is due to the small rates of population change within the study period (Table 1). To clearly illustrate the utility of ST-DB we carried out a simulation study with two different scenarios of rapidly changing background population (concentrated vs dispersed population increase). The results of the simulation study demonstrate that the spatiotemporal distribution of the background does have a substantial effect on the resulting risk estimates. This effect is visually detectable and apparent from Fig. 11. In addition, the distribution of the background has an effect on the benefit of considering the temporal dimension in our analysis: In both simulation scenarios, ST-DB produces higher odds ratios than S-DB across all parameter configurations, where the difference between odds ratios increases toward extreme parameter values. However, while differences increase toward higher percentile and lower *support* threshold values in the concentrated scenario, they increase toward higher percentile and higher *support* in the dispersed scenario. This shows us which parameter configuration we may choose to maximize the benefit of considering the temporal dimension of our data for different distributions of the background.

The comparison between space–time kernel density estimation for dynamic (ST-DB) and static (ST-SB) backgrounds underlines the usefulness of ST-DB. Health officials may be interested in the ability to identify areas that conform to the expectation of *high population density equals high case density* versus areas that do not. High risk areas/times identified through ST-DB could be targeted for interventions like mosquito control or awareness campaigns in the case of dengue fever.

The results obtained here point toward the following weaknesses and discussion points, some of which need to be addressed in the future. First, the observed population change in the city of Cali was moderate, therefore we simulated two high population growth scenarios to show how ST-DB performs under such conditions. However, more simulation studies are needed to demonstrate the benefit of considering the temporal dimension, e.g., under a population decrease scenario. Second, ST-DB assumes that cases and population are distributed on an infinitely continuous planar space, which justifies the use of Euclidean distance. However, as people and goods move along the road network, it is necessary to adapt ST-DB toward network distance, drawing from existing research about kernel density estimation for networks (Okabe et al. 2009), space–time hotspot detection for street-level incidents (Shiode and Shiode 2013), and local indicators of network‐constrained clusters (Yamada and Thill 2007). Third, we use epidemiological data under the assumption that people contracted the disease at their residential location. However, this is not necessarily true, as people move around the city for daily commutes or leisure time activities. Therefore, uncertainty in the spatiotemporal location of disease cases could undermine our results. Lastly, our use of the ‘people-days’ metric to quantify the population within the kernel may face the following weakness: a given number of people-days may be achieved by multiple ways that represent different conditions for disease transmission. For instance, 12 people present in a kernel for one hour each has the same result like on person present for 12 h. Hence, domain knowledge is key to establish a measure that best suits the purpose at hand.

We think that ST-DB is most useful for scenarios where the phenomenon of interest, as well as the background varies considerably in space and time, which depends on the scale and spatiotemporal extent of the study area/period, as well as data availability. Analyzing infectious disease at the municipal or regional level is a prime example of such a situation. Other application examples include chronic disease in rapidly urbanizing regions, or traffic accidents under varying traffic volumes. On the other hand, we think that ST-DB is likely not useful if the background is relatively static. For instance, many western cities are at a stage in their development where population density has not changed substantially for years, which would diminish the benefits of ST-DB. Lastly, while ST-DB is suitable for municipal- or regional-level analyses over multiple years, it is less suitable for global- or continental-level analyses over decades, because spatiotemporal data tend to be coarse at such scales, thereby reducing variation in geographic phenomena across space and time.

## Conclusions

In this study, we presented ST-DB, a kernel density estimator for spatially *and* temporally dynamic background populations. Our approach demonstrated the utility of accounting for a dynamic background for kernel density estimation by comparing it to an estimator that ignores the temporal dimension (S-DB). To the best of our knowledge, this approach is the first within the GIScience domain that explicitly accounts for a changing background population through time. We showed that accounting for temporal variation in the background in addition to spatial variation (ST-DB) may produce stronger clusters (as expressed in odds ratios) than an approach that only accounts for spatial variation (S-DB) for some parameter configurations.

Our approach addresses the limitations of current methods by adjusting kernel density estimates for dynamic backgrounds. We need to revisit and update current methods for analyzing spatiotemporal phenomena to explicitly incorporate temporal variation in background population. In the face of current migration and urbanization trends, as well as the availability of population data at high spatiotemporal resolution, this step has been long overdue.

Future work includes various improvements and applications of kernel density estimation for spatially and temporally dynamic backgrounds: first and foremost, it should be a priority to address some of the limitations mentioned here, including additional simulation studies to confirm the general validity of our findings. Second, we plan to develop a parallel version of ST-DB, which allows harnessing the processing power of high-performance computing and increases applicability of the method. Third, new methods for kernel density estimation under dynamic backgrounds are needed, which are able to accommodate various formats and conceptualizations of population data (Fig. 1). This will require further thinking about issues of scale and granularity of spatiotemporal analyses, as data types range from individual trajectories to population counts that are aggregated to the spatiotemporal units of census tracts and decades.

## Data Availability

The dengue fever dataset cannot be made publicly available as we do not have an agreement with the provider to share it. A detailed description of its content and format, which should be sufficient for applying ST-IB to other spatiotemporal point datasets, as well as all other data and codes used in this study are available in [https://figshare.com/s/7ff3db21fe2a10a39356].

## Footnotes

1 Note that we would need to increase the number of simulations substantially to achieve confidence levels acceptable in health studies (1,000 – 10,000 simulations).

## Code availability

All codes for this research are available in [https://figshare.com/s/7ff3db21fe2a10a39356].
